# Massage treatment of hyperplasia of mammary glands: A protocol for a systematic review and meta-analysis

**DOI:** 10.1097/MD.0000000000023601

**Published:** 2020-12-24

**Authors:** Dehui Ma, Guochao Liu, Xiaolin Zhang, Qi Zhang, Tianjiao Gao, Mingjun Liu

**Affiliations:** a Changchun University of Chinese Medicine; b The Changchun Hospital of TCM, Changchun, Jilin, China.

**Keywords:** hyperplasia of mammary glands, massage, meta-analysis, systematic review

## Abstract

**Background:**

With the accelerated pace of life, the problems of residence, diet, and environment have occurred frequently in recent years. External factors are easily to cause endocrine disorders and hormone sensitivity of breast tissue, which can lead to mammary hyperplasia. The incidence rate of hyperplasia of mammary glands is increasing year by year, and the age of onset is also getting lower and lower. If not treated in time, there is a crisis of breast cancer.

Clinical studies have found that massage is widely used in clinical treatment of mammary hyperplasia recently, but the efficacy of massage in the treatment of mammary hyperplasia has not been systematically reviewed. The purpose of this study is to explore the efficacy, safety and effectiveness of massage in the treatment of hyperplasia of mammary glands.

**Methods:**

We will search PubMed, Cochrane Central Register of controlled trials (central), ScienceNet, EMBASE, CBM, CNKI, VIP and Wanfang databases. The retrieval date was October 20, 2020. RevMan 5.3 software was used to evaluate the quality and risk of included studies. The efficacy, recurrence rate, and symptom score of breast hyperplasia were analyzed, and the results were observed and measured.

**Results:**

This study will be from the clinical efficiency, improvement rate, pain symptoms disappear rate, tumor size improvement rate and other aspects of the existing evidence for a high quality synthesis, as well as massage adverse events.

**Conclusion:**

the conclusion of this review will provide the basis for judging whether massage is safe and effective in the treatment of hyperplasia of mammary glands.

**Ethics and dissemination:**

This systematic will evaluate the effectiveness and safety of massage in the treatment of hyperplasia of mammary glands. As all data used in this systematic review and meta-analysis have been published, ethical approval is not required for this review.

**Protocol registration number:**

INPLASY2020100066

## 1 Introduction

Hyperplasia of mammary glands is a degenerative disease and progressive connective tissue growth caused by hyperplasia of mammary fiber and epithelial tissue.^[1]^ Studies show that breast hyperplasia is the highest incidence rate of female breast diseases, and has a certain correlation with the menarche time, the number of fetal birth, social economic status, and education level. About 75% of women have a certain degree of breast hyperplasia, and about 20% of women will be troubled by their clinical symptoms, among which 25 to 45 years old women have the highest incidence rate.^[2]^ the clinical manifestations of postmenopausal women were obvious atrophy of glands and aggravation of cystic lesions. Atypical hyperplasia is a precancerous lesion. The incidence rate of breast hyperplasia is also increasing with the increase of the disease course. According to the literature statistics, the canceration rate is between 1.25% and 50% then the HMG is a global health problem for women_._^[3]^

As one of the external treatment methods of traditional Chinese medicine, massage has been widely used in clinic because it can harmonized yin and Yang, promoting blood circulation and Qi, dredging meridians and collaterals, dredging collaterals and relieving pain, dissipating stasis, and dredging collaterals. It has obvious curative effect, convenient operation, low risk, non-toxic side effects, and is easily accepted by patients.^[4–12]^ In the treatment of mammary gland hyperplasia, hormone drugs, and iodine preparations are mainly used in reducing the pain of patients.^[13]^ It believes that the physical stimulation of massage manipulation causes changes in biophysics and biochemistry in the action area, and physiological reactions occur in local tissues in modern medicine.^[14]^ Through the regulation of nerve reflex and fluid circulation, this reaction is strengthened. On the other hand, it causes the overall secondary reaction, thus producing a series of changes in pathophysiological process and achieving therapeutic effect of benefitting lungs and soothe liver, regulating Yang, and activating blood circulation.^[15]^ Massage can significantly improve the degree and frequency of breast pain, inhibit, or reduce the volume growth of breast mass. However, there is no systematic review on massage treatment of hyperplasia of mammary glands. This is the first time to use meta-analysis randomized controlled trial (RCT) to explore the effect of massage intervention on patients with mammary hyperplasia.

## 2 Methods

The protocol has been registered on the INPLASY website, and the registration number is INPLASY2020100066) (https://inplasy.com/inplasy-2020-10-0066/). The ethical approval and patient informed consent are abandoned because this study is based on published or registered RCTs.

### 2.1 Inclusion criteria for study selection

#### 2.1.1 Type of studies

Only RCTs are included in our studies. Other designs, such as in vivo, in vitro, case reports, retrospective studies, and non-RCTs will be excluded. There are no restrictions on languages.

#### 2.1.2 Types of participants

Participants met the clinical diagnostic criteria for breast hyperplasia and were not lactating women; patients and their families informed the study and signed the consent form. At the same time, there are no restrictions on age, gender, region, nationality, and nationality. Cases associated with serious illness, pregnancy, and drug-induced obesity were not included.

#### 2.1.3 Types of interventions

Observation group: the intervention time of massage therapy was at least 4 weeks. Massage therapy was stopped during menstruation, and the treatment time was postponed.

Control group: mainly used oral medicine or placebo or other traditional Chinese medicine. However, it be combined with other treatments during the treatment.

#### 2.1.4 Types of outcome measures

The main criteria were: complete disappearance of pain symptoms; diameter and area of breast mass; hormone levels of luteinizing hormone, 17α-estradiol, prolactin, and progesterone The secondary outcome measures were abnormal menstruation and mood changes. At the same time, observe whether there are adverse reactions or adverse events in the treatment process to comprehensively evaluate the clinical efficacy and safety of massage in the treatment of breast hyperplasia.

#### 2.1.5 Data source

##### 2.1.5.1 Electronic searches

Database search includes: Search PubMed, Cochrane Central Register of Controlled Trials (CENTRAL), Web of Science, EMBASE, CBM, CNKI, VIP, and Wanfang databases were searched by computer. The retrieval date was up to October 20, 2020. According to the requirements of different databases, the corresponding retrieval format is adopted. In order to avoid omission, the search scope includes subject words, key words or full text. The Chinese key words include “hyperplasia of mammary gland”, “hyperplasia of mammary gland”, “massage” and so on, while the English key words are “hyperplasia of mammary gland”, “hyperplasia”, “Tuina” and “massage”. The search term in the Chinese database is the translation of the above word. The complete PubMed search strategy is summarized in Table 1.

**Table 1:** Search strategy used in PubMed database.

Number	Search terms. Ti, ab.
1	Craniosacral massage. Ti, ab.
2	Massage, craniosacral. Ti, ab.
3	Zone therapy. Ti, ab.
4	Therapies, zone. Ti, ab.
5	Zone therapies. Ti, ab.
6	Therapy, zone. Ti, ab.
7	Reflexology. Ti, ab.
8	Rolfing. Ti, ab.
9	Bodywork. Ti, ab.
10	Bodyworks. Ti, ab.
11	Massage therapy. Ti, ab.
12	Massage therapies. Ti, ab.
13	Therapies, massage. Ti, ab.
14	Therapy, massage. Ti, ab.
15	or 1–14
16	Period, postpartum. Ti, ab.
17	Postpartum. Ti, ab.
18	Postpartum women. Ti, ab.
19	Women, postpartum. Ti, ab.
20	Puerperium. Ti, ab.
21	or 16–20
22	Appetite depressants. Ti, ab.
23	Body weight. Ti, ab.
24	Diet, reducing. Ti, ab.
25	Skinfold thickness. Ti, ab.
26	Lipectomy. Ti, ab.
27	Anti-Obesity agents. Ti, ab.
28	Bariatrics. Ti, ab.
29	or 22–28
30	Randomized controlled trial. Pt.
31	Controlled clinical trial. Pt.
32	Randomized. Ab.
33	Randomly. Ab.
34	trial. Ab.
35	or 30–34
36	exp animals/not humans.sh.
37	35 not 36
38	15 and 21 and 29 and 37

##### 2.1.5.2 Searching other resources

The manual search mainly is used for searching relevant studies, such as “China Journal of Traditional Chinese Medicine and Pharmacy”, “Journal of Acupuncture and Tuina Science”.

### 2.2 Data collection and analysis

#### 2.2.1 Selection of studies

According to the inclusion and exclusion criteria, 2 researchers independently screened the literatures. In case of disagreement, they were discussed or decided by a third party. First, according to the inclusion and exclusion criteria, 2 researchers (G-TJ and L-GC) independently selected the literature after reading the title and abstract. Second, by reading the full text, we exclude uncontrolled studies, inconsistent evaluation criteria, and similar data. If there is any difference during the screening study, the third author (ZQ) will be involved.

#### 2.2.2 Data extraction and management

**Figure 1:**
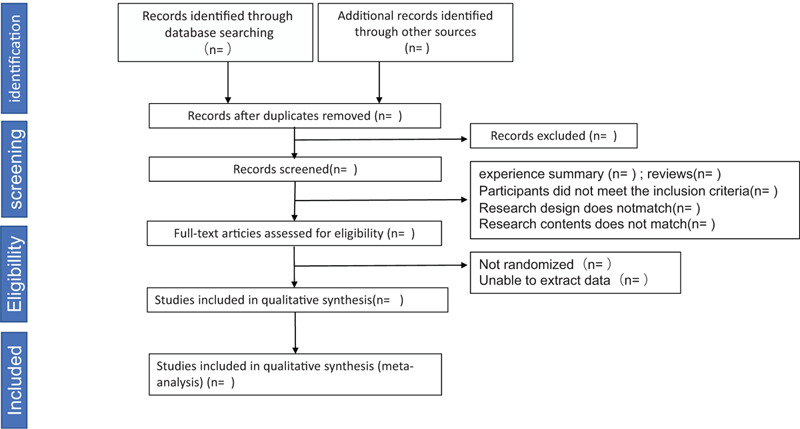
the PRISMA flow chart.

Two researchers (G-TJ and L-GC) used a pre-designed data extraction table to extract the final included data, including the author, year, sample size, treatment process, intervention measures, outcome indicators, adverse reactions, etc. The process of study selection will be carried out using the flow chart shown in the PRISMA flow chart (Fig. 1).

### 2.3 Statistical analysis

We will use RevMan 5.3 software for Mean difference was used as measurement data, odds ratio (or) was used as measurement index for categorical variables, and 95% confidence interval was used as effect quantity.^[16]^ Firstly, the statistical heterogeneity of the included clinical RCTs was analyzed by Cochrane I^2^ test. When I^2^ < 50% or *P* > .05 indicated that there was no statistical heterogeneity among the studies, the fixed effect model was selected to combine the effect amount; otherwise, the random effect model was adopted. The therapeutic effect of massage in the experimental group was compared with that in the control group by forest map, and the sensitivity analysis was carried out according to the main aspects that may lead to clinical heterogeneity to identify the source of heterogeneity.

#### 2.3.1 Methodological quality of assessment

The literature quality of this study was assessed using the bias risk table recommended by the Cochrane Collaboration. The risk table includes 6 items: random sequence generation method, allocation concealment, blinding of subjects, and intervention providers, blinding of outcome evaluators, completeness of result data, selective result reporting and other sources of bias. The criteria for assessing the risk of bias were “low risk”, “high risk” and “unclear”. In this process, 2 reviewers independently evaluate the quality of methodology. In case of disagreement, a third party will be invited to make a decision.

#### 2.3.2 Assessment of heterogeneity

Further analysis can be performed using the I^2^ test. If possible, we will also build a forest map for analysis. There was no significant heterogeneity (I^2^ < 50%) between a group of studies, and the fixed effect model was used to evaluate. If there is significant heterogeneity between a group of studies (I^2^ > 50%). We will explore the reasons for the existence of heterogeneity from various aspects such as the characteristics of the subjects and the degree of variation. The source of heterogeneity is further determined by means of sensitivity analysis.

#### 2.3.3 Assessment of publication bias

If the results of meta-analysis include more than 10 articles, we can use Revman5.3 software to draw and analyze funnel chart, and check whether there is publication deviation in this study by drawing funnel map. Funnel map symmetry means that there is no publication offset, and asymmetry is the opposite.

#### 2.3.4 Quality of evidence

The evidence quality of the main results will be assessed by the proposed grading assessment and the development of evaluation methods. Evaluation includes bias risk; heterogeneity; indirectness; imprecision; publication bias.

#### 2.3.5 Grading the quality of evidence

We will summarize the GRADE judgements according to the Grading of Recommendations Assessment, Development, and Evaluation^[17]^ method to analyze the quality level of evidence.

## 3 Discussion

Oral drugs, especially hormone drugs, have certain side effects in the treatment of mammary hyperplasia at present. Western medicine or Chinese patent medicine often stimulate the gastrointestinal tract, and long-term medication will produce adverse reactions. Patients often relapse due to daily factors such as high pressure, poor sleep, mood fluctuation, and so on, and the recurrence rate is high. Although the acupuncture and catgut embedding therapy are effective, there are still some limitations in patients’ fear of acupuncture and syncope.^[18]^ Therefore, to explore more effective and safe treatment and improve the prognosis of patients with mammary hyperplasia has become an important health problem. As an external treatment method of traditional Chinese medicine, massage has a good therapeutic effect on hyperplasia of mammary glands.^[19]^ It can be used to relax the tendons and disperse the knot, so that the meridians can be infused normally and promote the blood circulation. It has the advantages of rapid onset, simple and convenient, short course of treatment, safe and reliable, and easy to be accepted by patients.

Since there is no systematic meta-analysis on the efficacy of massage in the treatment of hyperplasia of mammary glands, it is hoped that these results can provide clinicians with the basis of massage in the treatment of mammary hyperplasia, and provide high-quality basis for the effectiveness and safety of massage in the treatment of mammary hyperplasia.

Due to the inability to search all databases and unpublished studies, there may be incomplete literature. Moreover, this study only includes Chinese and English literature, which may lead to selection bias. This study can provide the basis for the related research of massage treatment of mammary hyperplasia.

## Author contributions

**Conceptualization:** Mingjun Liu, Dehui Ma

**Data curation:** Xiaolin Zhang, Tianjiao Gao, Guochao Liu, Qi Zhang.

**Data synthesis:** Tianjiao Gao, Xiaolin

**Formal analysis:** Qi Zhang.

**Methodology:** Dehui Ma, Qi Zhang

**Software:** Mingjun Liu, Dehui Ma

**Supervision:** Mingjun Liu.

**Writing – original draft:** Dehui Ma, Guochao Liu.

**Writing draft:** Mingjun Liu, Dehui Ma, Guochao Liu, Qi Zhang
